# Asking the right questions: How PMS question phrasing impacts responses in an English speaking, online sample

**DOI:** 10.1007/s00737-025-01598-7

**Published:** 2025-09-01

**Authors:** Gabriella Kountourides, Alexandra Alvergne

**Affiliations:** 1 School of Anthropology and Museum Ethnography, University of Oxford, 51, 53 Banbury Rd, Park Town, Oxford, OX2 6PE UK; 2 ISEM, Univ Montpellier, CNRS, IRD, Pl. Eugène Bataillon, Montpellier, 34090 France

**Keywords:** Premenstrual change, Sentiment analysis, PMS, Framing, Bayesian statistics

## Abstract

**Purpose:**

The discourse around menstrual cycles is often pathologized, potentially reinforcing negative perceptions of menstruation. The extent to which individuals have internalized the idea that bodily and social experiences before menstruation are the manifestation of ill-health, thereby biasing reports of premenstrual experiences towards negative emotions, remains unclear.

**Methods:**

Using an online experimental design, we investigate whether phrasing the premenstrual experience as having both negative and positive dimensions would enable individuals to report more diverse and positive experiences than are reported in the absence of specific emotional prompts. Participants were recruited using a period tracker app and randomly allocated to one of three conditions: control (describe your premenstrual experience); treatment 1 (describe your negative and positive premenstrual experience); treatment 2 (describe your posititive and negative premenstrual experience). Sentiment analysis was used to derive polarity scores, and a two-part Bayesian model assessed the impact of phrasing order.

**Results:**

Among 2,637 participants, responses skewed negatively (mean -0.25). Compared to the control, treatment conditions 1 and 2 reported premenstrual experiences 64% and 62% less negative, respectively. Positive themes, notably ‘sex, libido, and energy’emerged. The absence of positive prompts in questioning led to more negative and less diverse reports.

**Conclusions:**

These findings support existing literature on the predominance of negative premenstrual phases and underline the need to broaden measurements to encompass positive symptoms. The study also pioneers the use of text analysis for investigating premenstrual symptoms.

## Article Highlights

• Large Sample Size: We use data from 2638 English-speaking women recruited through the Clue period tracker app; this study represents the largest of its kind, providing valuable insights into the premenstrual experience.

• Negative Reporting Bias: We find a default tendency among women not to report positive premenstrual experiences unless specifically prompted, suggesting the influence of language and social constructs on how menstruation is described.

• Impact on Measurement Approaches: By challenging the prevailing notion of exclusively negative premenstrual experiences, the study calls for a re-evaluation of measurement approaches in women's health research, advocating for a more balanced perspective.

• Innovative Text Analysis: Our application of text analysis to capture both positive and negative polarity in women's accounts of the premenstrual experience opens up new avenues for understanding and characterizing women's health, paving the way for future research in the field.

## Introduction

The premenstrual phase is usually portrayed in medical discourse and educational contexts as being associated with a state of ill-health (Walker et al. 1998; Heard et al. 1999; Chandra and Chandra 1989), which has been argued to influence women’s expectations of their menstrual cycle and bias reports of the premenstrual phase towards a negative experience. This phenomenon is thought to arise from the medicalization of the female body in Western biomedicine (Rose 1994), whereby normal reproductive processes are redefined as diseases, as is observed when the premenstrual phase becomes synonymous with premenstrual syndrome (PMS). While PMS has been linked to various biological mechanisms and is possibly driven by cyclical inflammation changes during the menstrual cycle (Puder et al. 2006; Bertone-Johnson et al. 2014; Bertone-Johnson 2016; Alvergne and Högqvist Tabor 2018; Mattina et al. 2019), the extent to which people have internalized the premenstrual phase as a syndrome, i.e., an experience perceived as more negative than it actually is, remains unclear. This paper aims to address this gap by using text analysis to compare the emotional valence and diversity of premenstrual reports in the absence and presence of emotional prompts.

PMS, the physical or mood changes in the 5 days before a period starts (Winer and Rapkin 2006), affects approximately 48% of fertile women worldwide according to the American College of Obstetricians and Gynecologists (American College of Obstetricians and Gynecologists 2015; 2023). Other studies put this figure closer to 91% (Tschudin, Bertea, and Zemp 2010). For some, PMS can be debilitating and can interfere with everyday life, both at work (Heinemann et al. 2010) and home (Rapkin and Winer 2009). However, beyond bloating, fatigue, headaches, and mood changes (Direkvand-Moghadam et al. 2014), women also report positive and potentially healthy correlates, such as heightened creativity (Campagne and Campagne 2007; King and Ussher 2013; Martin 1987), an increase in energy (Halbreich et al. 1982; Altmann, Knowles, and Bull 1941; Abplanalp, Donnelly, and Rose 1979; Alagna and Hamilton 1986), increased libido (Halbreich et al. 1982), and describing feeling “on top of the world” (Woods et al. 1992). Premenstrual health is assessed through questionnaires primarily measuring the negative symptoms of the premenstrual phase (Knaapen and Weisz 2008; Romans et al. 2012). These psychometric tools, of which up to 60 are available, have been criticized for assuming that women’s reproductive health is solely determined by biology, disregarding women’s voices and lived experiences (Taylor 2006).

Menstrual-related mood changes may in part stem from the internalization of a cultural construct linking negative mood with the premenstrual phase (Clarke and Ruble 1978; Olasov and Jackson 1987; Sommer 1973; Parlee 1974). Completing a menstrual joy questionnaire, rather than a Menstrual Distress Questionnaire (MDQ), increased women’s score of positive arousal when considering cycle-related symptoms (Rose, Chrisler, and Couture 2008). A study of 50 women in the US found that participants could be primed to adopt more positive perspectives towards menstruation, regardless of their previous menstrual experiences (Chrisler et al. 1994). Conversely, a negative bias in questionnaire items could result in negative responses and potentially contribute to overall negative attitudes. Being aware that a study is menstrual-related leads to more reports of depressed mood and fatigue, a result which has been replicated in various samples (Sommer 1973; Parlee 1974; Aubuchon and Calhoun 1985). An American study involving 112 women not using hormonal contraception found that using short lectures to modify negative menstrual and premenstrual phase expectancies (to increase or decrease negative expectations) succeeded in altering negative expectancies up to 40 days after the study (Olasov and Jackson 1987). In the study, women shown a video describing a connection between the menstrual cycle and low mood increased their expectations of negative mood changes and diminished their expectations of positive changes (Olasov and Jackson 1987). However, studies that have investigated the influence of question phrasing on the sentiments associated with PMS reports were typically conducted on relatively small sample sizes (n < 100) and did not consider neutral prompts, thus limiting our ability to infer if and when women have internalized negative medicalized views of PMS in the absence of any specific prompt (i.e., by default).

Here, we aimed to evaluate whether women who menstruate have internalized overly negative perceptions of their premenstrual phase through analyzing textual reports of the premenstrual experience. We used an online experiment to manipulate question phrasing (neutral vs. emotionally polarized). We compared premenstrual testimonies across experimental conditions for their polarity (a measure of how positive and/or negative text is), emotional intensity (i.e., the magnitude of polarity), and textual diversity.

## Materials and Methods

### Recruitment

This study received ethical approval from the Research Ethics Committee of the School of Anthropology and Museum Ethnography (SAME_C1A_19_07). Participants were recruited through push notifications to 30,000 randomly selected users of the period-tracker app Clue (‘Clue.’ 2017) whose app language was set to English. The survey was hosted on Qualtrics. The response rate was 8.6%. Participants were over 18 and had a menstrual bleed within the past three months.

### Survey

The survey included a maximum of 21 questions, with the exact number of questions depending on individual circumstances. We collected data on age, level of partner support, medication usage, country of birth, vitamin supplementation, and contraceptive usage. Qualitative reports of the premenstrual experience were gathered through a free text entry question (SI.1). Data collection took place from the 13/03/2020 through to 01/07/2020 and was halted once no new responses had been recorded for a consecutive three-day period.

### Experimental design

**Fig. 1:**
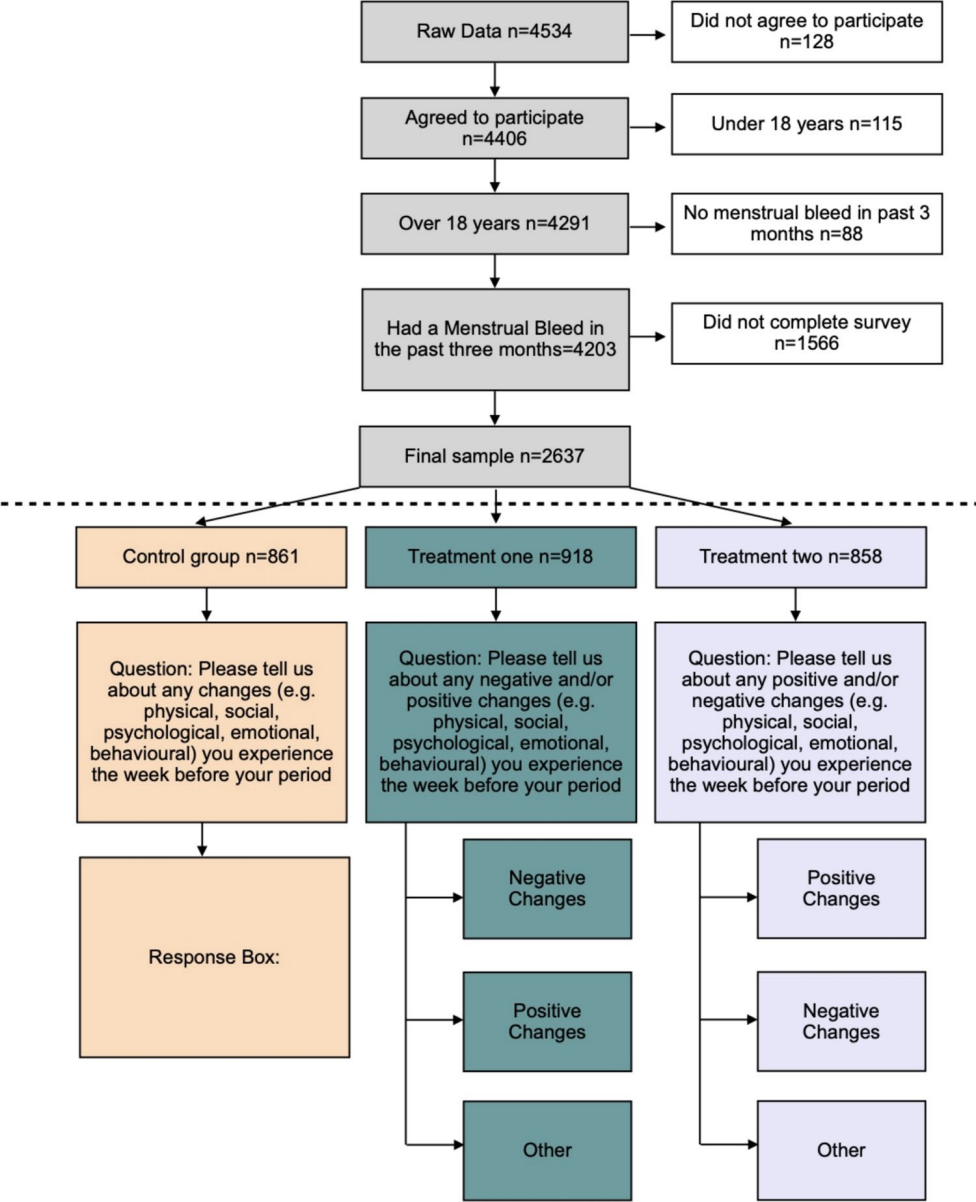
Flowchart of sample selection and treatment allocation. Individuals under 18, those who did not consent to participate, those who had not experienced a menstrual bleed in the past 3 months or did not complete the survey were excluded. The remaining 2,637 participants were randomly assigned to one of three conditions, which differed in the wording of the question asking them to describe their premenstrual experience

Participants were randomly assigned to one of three conditions, differing in the wording of the question that asked participants to describe their premenstrual experience. Participants in the control condition were exposed to a neutral wording (“Please tell us about any changes (e.g., physical, social, psychological, emotional, behavioral) you experience the week before your period.”) and provided their responses in a single text box. Participants in the treatment conditions received modified versions of the question, replacing “any changes” with “any positive and/or negative changes” (treatment 1) or “any negative and/or positive” changes” (treatment 2)(Fig. 1). Both treatments were answered using three separate boxes: ‘negative’, ‘positive’, and ‘other’, (with ‘positive’ and ‘negative’ reversed for treatment 2). The terms ‘PMS’ or ‘premenstrual syndrome’ were avoided to prevent any potential bias.

### Text Analysis

#### Data cleaning

Text processing was performed prior to analysis to address common issues such as unedited text, typos, and abbreviations that are encountered in questionnaire data (Wan and Gao 2015). A cleaning function was adapted from the textclean package (Rinker 2018) to remove heterogeneity in wording (e.g., PMS, premenstrual syndrome).

#### Polarity scores

To create a polarity score for each participant’s response, we performed sentiment analysis using the sentimentr package (Rinker 2019). The sentimentr package uses a pre-defined dictionary (lexicon) of words and assigns positive, negative, and neutral tags to sentences based on the presence of these words. The algorithm considers negators, shifters, and intensifiers (e.g., not and really) to calculate the final polarity score (Mostafa 2013). We created a custom dictionary adapted to menstruation by editing the polarity of relevant words in the default lexicon of the sentimentr package (SI.2). Words that described ‘no experience’, such as ‘none’, ‘nil’, or ‘NA’ were recoded as ‘none’ and a neutral score of 0 was applied. After checking for the efficacy of the algorithm by comparing the polarity of responses in ‘positive’ and ‘negative’ boxes using McNemar’s ${\chi }^{2}$, textual data pertaining to each participant’s response was combined for statistical analysis. The analysis does not ‘cancel out’ negative and positive but considers sentences and provides an overall score.

#### Word diversity

The data cleaned for the previous analysis underwent further processing by tokenizing the text resulting in a one-word-per-row format (two words for bigrams). Stopwords that do not carry significant meaning, such as “and” and “thus”, were removed from the text (SI.3). Lemmatization was applied to condition together various forms of the same word (e.g., hunger, hungry), using the textstem package (Rinker 2018) (SI.4). The accuracy of the algorithm was assessed (SI.5).

#### Word frequency

We identified the 10 most frequently used singular words in each of the three conditions.

#### Lexical density

For each condition, the ratio of unique words to the total number of words was calculated. Higher lexical density scores indicate a greater variety of unique words and less repetition within the conditions’ responses.

### Statistical Analysis

Missing data imputation was conducted to address the presence of incomplete cases using the missRanger (Mayer 2021) package. The final models were run five times, each on one of the imputed datasets, and the estimates pooled. To check for imputation accuracy, the models were run on both the complete and the imputed datasets (SI.6).

To account for the occurrence of zeros (neutral responses) when modelling polarity, we used a two-part model (Neelon et al. 2016). We used a multinomial logistic regression model to analyze the polarity of responses grouped into positive (> 0), negative (0 <), and neutral (0) (model 1). We used minimally informative priors, with the negative polarity group as the reference category. We used a continuous regression model fitted with a Gamma distribution to analyze the range of negative polarity scores (model 2), which were inverted to use a continuous probability distribution, and the range of positive polarity scores (model 3). Age was standardized.

Models were fitted using Stan computational framework (Carpenter et al. 2017) and the Hamiltonian Monte Carlo algorithm (Bürkner 2017). Uncertainty in the estimates was summarized using 95% credible intervals. Model comparison was performed using the leave-one-out cross-validation (LOO-CV) method (Gabry et al. 2019). To assess the relative fit of the full model, a null model was compared using an approximate LOO-CV approach (SI.7) (Vehtari et al. 2017). Post-predictive checks can be seen in (SI.8). Traceplots showed that the MCMC chains had achieved both stationarity and effective mixing, supporting model convergence (SI.9) (McElreath 2018).

## Results

A total of 4,534 individuals participated in the study. After removing unfinished responses and those that did not meet the inclusion criteria, the final sample size included 2,637 participants (Fig. 1). Participants’ ages ranged from 18 to 54 years, with a mean age of 28 years. The sample includes participants born in 117 different countries, with the majority being born in the UK (n = 713), the United States (n = 590), Australia (n = 248), and Canada (n = 113). Among the participants, 17% reported using hormonal contraception (n = 457), a proportion that aligns with the anticipated global rate of hormonal contraceptive use (21.5% (United Nations 2019) (Table 1).

**Table 1:** Characteristics of Treatment Conditions. All quotes are provided with consent

	***Control Condition***	***Treatment Condition 1***	***Treatment Condition 2***
***Background Characteristics***			
*Participants per condition*	861	918	858
*Age (mean* + *sd)*	28.1 (7)	28.2 (6.8)	28.2 (6.8)
*Residence in the UK*	235 (27.3%)	238 (25.9%)	240 (28%)
*Hormonal Contraceptive Use*	152 (17.7%)	165 (18%)	140 (16.3%)
***Area of Residence***			
*Africa*	14 (1.6%)	8 (0.9%)	13 (1.5%)
*Asia and the Pacific*	70 (8.1%)	64 (7%)	49 (5.7%)
*Oceania*	80 (9.3%)	108 (11.8%)	93 (10.8%)
*Europe*	374 (43.4%)	361 (39.3%)	360 (42%)
*North America*	213 (24.7%)	266 (29%)	224 (26.1%)
*South America*	32 (3.7%)	29 (3.2%)	29 (3.4%)
***Polarity Scores***			
*Score (mean* + *sd)*	−0.4 (0.5)	−0.2 (0.3)	−0.2 (0.2)
*Range Score*	−4.25: 2	−1.15: 2	−1.04: 2
*Total Number of Negative Scores*	721 (83.7%)	675 (73.5%)	627 (73.1%)
*Total Number of Positive Scores*	121 (14.1%)	168 (18.3%)	157 (18.3%)
*Total Number of Neutral Scores*	19 (2.2%)	75 (8.2%)	74 (8.6%)
*Response Characteristics*			
*Number of Words per response (mean* + *sd)*	31.9 (39.3)	12.4 (11.6)	13.6 (13.3)
*Time taken to complete (mean* + *sd)*	4779.7 (121,191)	1038.7 (14,613.6)	511.9 (1525.8)
*Participant Response Box*			
*Text written only in"positive"box*	NA	22	18
*Text only written in"negative"box*	NA	466	389
*Text only written in"other"box*	NA	10	12
*Text in"positive"box*	NA	333	316
*Text in"negative"box*	NA	831	772
*Text in"other"box*	NA	137	197
*Text in"control"box*	852	NA	NA
*"None"written in"positive"box*	NA	585	542
*"None"written in"negative"box*	NA	87	86
*"None"written in"other"box*	NA	781	661
*"None"written in"control"box*	7	NA	NA
*Text scoring negative (score)*	"things i’ve noticed:—breasts feel sore/heavy—skin is oily and frequently have more pimples—hair becomes more oily—abdomen/stomach is heavily bloated/full—i feel more lethargic—i’ve noticed i am more prone to crying or feeling distressed"(−2.5)	"bad pms very very moody"(−2.3)	"Bloated, tired, sore breasts, irritable, tired, hungry, overly sensitive, cramping"(−2.1)
*Text scoring negative (score)*	"mood swings (quite drastic), generally more aggravated/emotional—sore/itchy boobs—anxiety increases (become especially socially anxious)—migraines/headaches—very sore hips/back—extreme fatigue—some cramping"(−4.24)	"moody, dramatic, emotionally, bitchy, overly sensitive, depressed, bloated"(−1.9)	"highly emotional, painful cramps, acne, dry skin, fatigue, sore muscles, sadness, anxieties, unhealthy cravings, bloating, clouded mind"(−1.4)
*Text scoring negative (score)*	"diarrhea, depression, cramping, crying spells"(−1.3)	"emotional, do not look forward to anything, feel miserable and hopeless, have cravings"(−0.9)	"uncontrollable mood swings, sore breasts, spotting, slight abdominal cramps"(−0.8)
*Text scoring positive (score)*	"higher sex drive social withdrawal weight gain"(0.9)	"more feminine energy (calm, nurturing, etc.)"(1.3)	"more motivation and energy"(1.4)
*"Text scoring positive"(score)*	"social- feel generally more empathetic, behavioral- motivated to do things such as cleaning or organization"(1.1)	"more in tune with my emotions"(0.7)	"urge to clean, urge to run, higher creative output, increased productivity"(0.9)
*Text scoring positive (score)*	"my legs feel weak and i become sexually aroused"(0.2)	"good skin, good hair, ginormous urge to clean and tidy up"(0.9)	"the day right before my period i get a burst of energy and i usually get so much done: cleaning, work, exercise, cooking large feasts, etc." (0.8)
*Text scoring neutral (score)*	"Nothing of note"(0)	"no other change"(0)	"Odd dreams"(0)
*Text scoring neutral (score)*	"i do not really notice any differences."(0)	"none"(0)	"my period has been constant since getting the implant"(0)
*Text scoring neutral (score)*	"Nothing much changed in the week before my period"(0)	"Just waiting for the release!"(0)	"more frequent outings into nature"(0)

### PME testimonies are overall negative

**Fig. 2:**
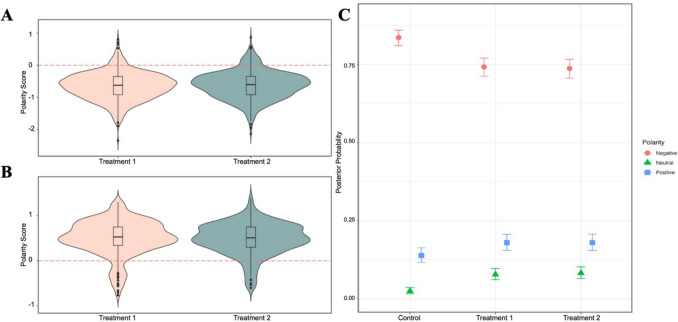
**Graph A and B**: Violin and Density plots illustrating score distribution, segregated by valence in treatment 1 (pink) and 2 (green). (A) shows the polarity score for text entered in the negative text boxes in the treatments, and (B) shows the polarity score for text entered in the positive text boxes in treatments 1 (pink) and 2 (green). All ‘neutral’ responses have been excluded. The red lines denote the 0 line, with negative polarity scores appearing below and positive polarity scores above it. The presence of only a limited number of responses on the opposite side of the 0 line indicates the sentiment analysis accurately assigns negative scores to responses written in the negative box with an 89% accuracy rate, and 78% accuracy for positive responses. The similarity in shapes between treatments indicates no significant difference in response scores between treatments 1 and 2. (C) shows the posterior probability of the polarity of PME reports, conditional on all other predictors. Error bars indicate 95% confidence intervals. Red circles: negative experiences; green triangles: neutral experiences; blue squares: positive experiences. Responses are generally negative across treatment conditions, but participants in treatment conditions one and two contributed more neutral responses, more positive responses, and fewer negative responses

The mean polarity of the text data was −0.25, indicating a negative skew across all three conditions (SI.10). The mean polarity score for the control condition was lower compared to the treatment conditions (control: mean (sd) = −0.4 (0.5), Treatment 1 (T1): −0.2 (0.3); Treatment 2 (T2): −0.2 (0.3), ${\chi }^{2}$=101.5, df = 2), a result confirmed by post-hoc pairwise comparisons (control vs. T1, p < 0.001; control vs. T2, p < 0.001). There was no effect of the order of emotional prompts on polarity score. In a Bayesian multinomial regression model adjusted for age and use of hormonal contraception, participants in treatment conditions had more than double the odds of reporting neutral rather than negative experiences as compared to those in the control condition (T1: OR = 3.58, 95% Credible Interval (CI):2.27–5.77; T2: OR = 3.81, 95% CI:2.41–6.16) (Fig. 2C, Table 2). Participants in the treatment conditions were also more likely to give positive responses (T1: OR = 1.46, 95% CI:1.14 −1.88; T2: OR = 1.47, 95% CI:1.14–1.9). A sensitivity analysis excluding individuals who may be experiencing perimenopause (age > 40) yielded similar results (SI.11). Polarity score was not associated with the type of contraceptive used (SI.12).

**Table 2:** Output of a multinomial regression model. The response variable is the polarity of responses with 3 categories (positive, neutral, and negative). ‘Negative’ was set as reference category. Bolded estimates indicate significant effects

	**Odds Ratio**	**Est.Error**	**l-95% CI**	**u-95% CI**
**Intercept [Neutral]**	**0.03**	**1.24**	**0.02**	**0.04**
**Intercept [Positive]**	**0.17**	**1.11**	**0.14**	**0.20**
**Treatment Condition 1: Neutral**	**3.58**	**1.27**	**2.27**	**5.77**
**Treatment Condition 2: Neutral**	**3.81**	**1.27**	**2.41**	**6.16**
Standardized Age: Neutral	1.02	1.09	0.87	1.20
Hormonal Contraception (True): Neutral	1.28	1.23	0.85	1.89
Treatment Condition 1: Positive	**1.46**	**1.14**	**1.14**	**1.88**
**Treatment Condition 2: Positive**	**1.47**	**1.14**	**1.14**	**1.90**
Standardized Age: Positive	0.92	1.06	0.82	1.02
Hormonal Contraception (True): Positive	1.14	1.15	0.87	1.48

### PME testimonies are more negative in the control condition

**Fig. 3:**
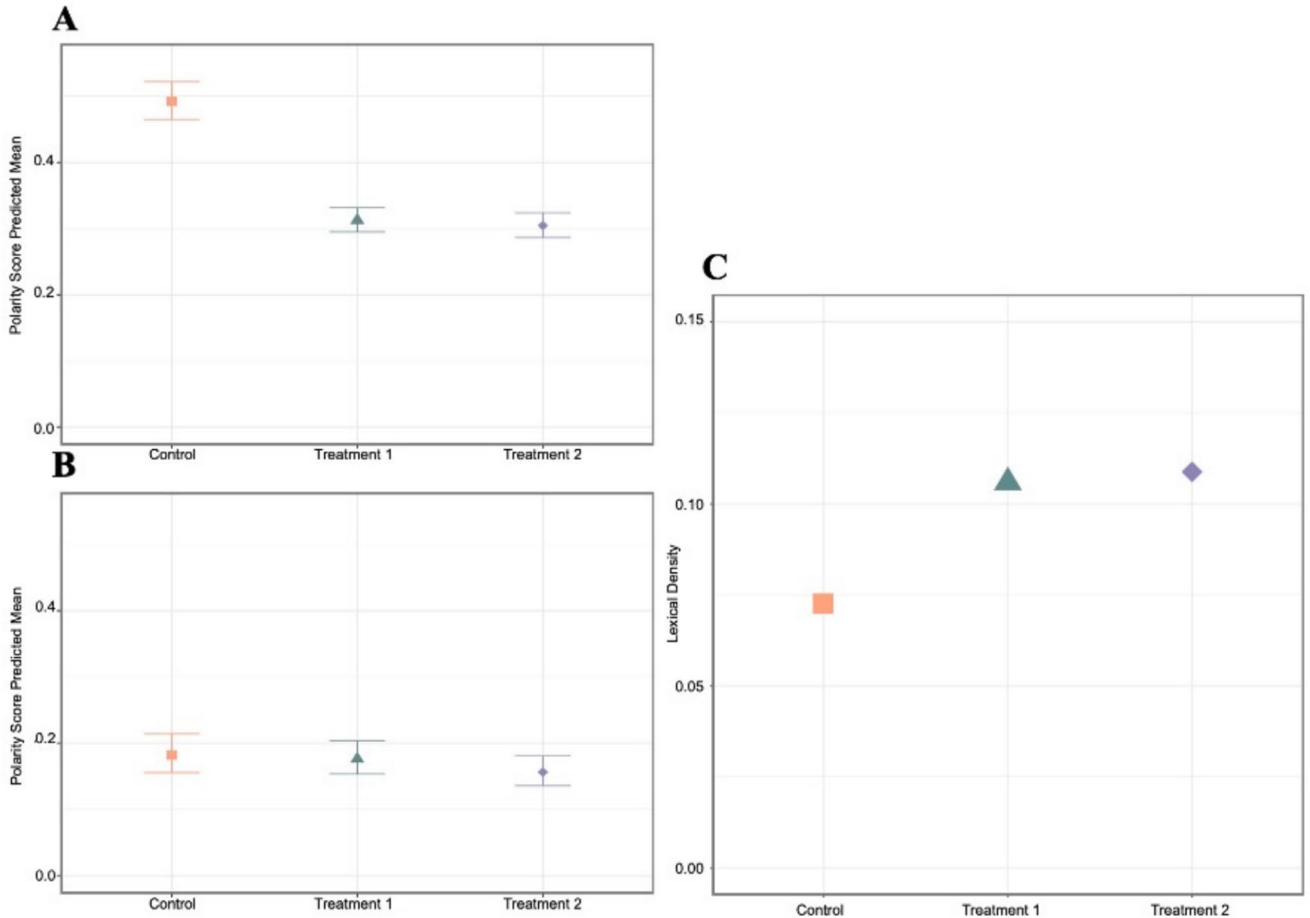
Predicted means of the magnitude of polarity (emotional intensity) of PME reports and its 95% Credible Intervals (A) negative polarity scores; (B) positive polarity scores. The small 95% credible intervals which do not overlap between control and treatments one and two in facet (A) suggests differences in the range of negative scores across experimental conditions. Larger 95% credible intervals in treatments one and two in facet (B) suggest less reliable differences in the range of positive responses. (C) Lexical density across treatment conditions. Lexical density corresponds to the ratio of unique words relative to the total number of words in each condition. Treatment conditions one and two contain significantly more unique words than the control condition

To examine the impact of question phrasing on emotional intensity for a given sentiment (e.g., how negative is a “negative” response?), we excluded neutral responses from the analysis. Within the subset of responses scoring negative, PME reports in treatment conditions were 64% less negative (T1: 95% CI:0.59–0.69) and 62% less negative (T2: CI:0.57–0.67) than those in the control condition (Fig. 3 A&B, Table 3). Within the subset of responses scoring positive, there were no differences across the experimental conditions (Table 3). Age and the use of hormonal contraception were not associated with the magnitude of polarity.

**Table 3:** Model output for the range of negative and positive polarity scores across experimental conditions. The estimates are the exponentiated estimate for the model. The estimate represents the multiplicative change in polarity score of a treatment compared to control. An individual in treatment 1 has a multiplicative change in strength of negative score. (a). Negative Model; (b). Positive Model. The control condition is the baseline. Bolded estimates indicate significant effects

	**Estimate**	**Est.Error**	**l-95% CI**	**u-95% CI**
**(a) Model on Negative Responses**				
**Intercept**	**0.49**	**1.03**	**0.46**	**0.52**
**Treatment Condition 1**	**0.64**	**1.04**	**0.59**	**0.69**
**Treatment Condition 2**	**0.62**	**1.04**	**0.57**	**0.67**
**Standardized Age**	**1.04**	**1.02**	**1.00**	**1.08**
Use Hormonal Contraception	0.99	1.05	0.90	1.08
**(b) Model on Positive Responses**				
**Intercept**	**0.18**	**1.09**	**0.16**	**0.21**
Treatment Condition 1	0.97	1.11	0.79	1.19
Treatment Condition 2	0.86	1.11	0.70	1.06
Standardized Age	1.01	1.04	0.93	1.10
Use Hormonal Contraception	0.91	1.11	0.74	1.13

### Symptom diversity is lower in the control condition

#### Most common words across conditions

Among all experimental conditions, both individual words (unigrams) and pairs of adjacent words (bigrams) in the negative treatment boxes exhibited a clear pain element (cramp, breast, and back pain) and emotional sensitivity. In contrast, positive boxes shared increased sex drive or libido across all three conditions, while only the treatments mentioned elements of enhanced energy, productivity and improved skin or hair.

##### Unigrams

**Fig. 4:**
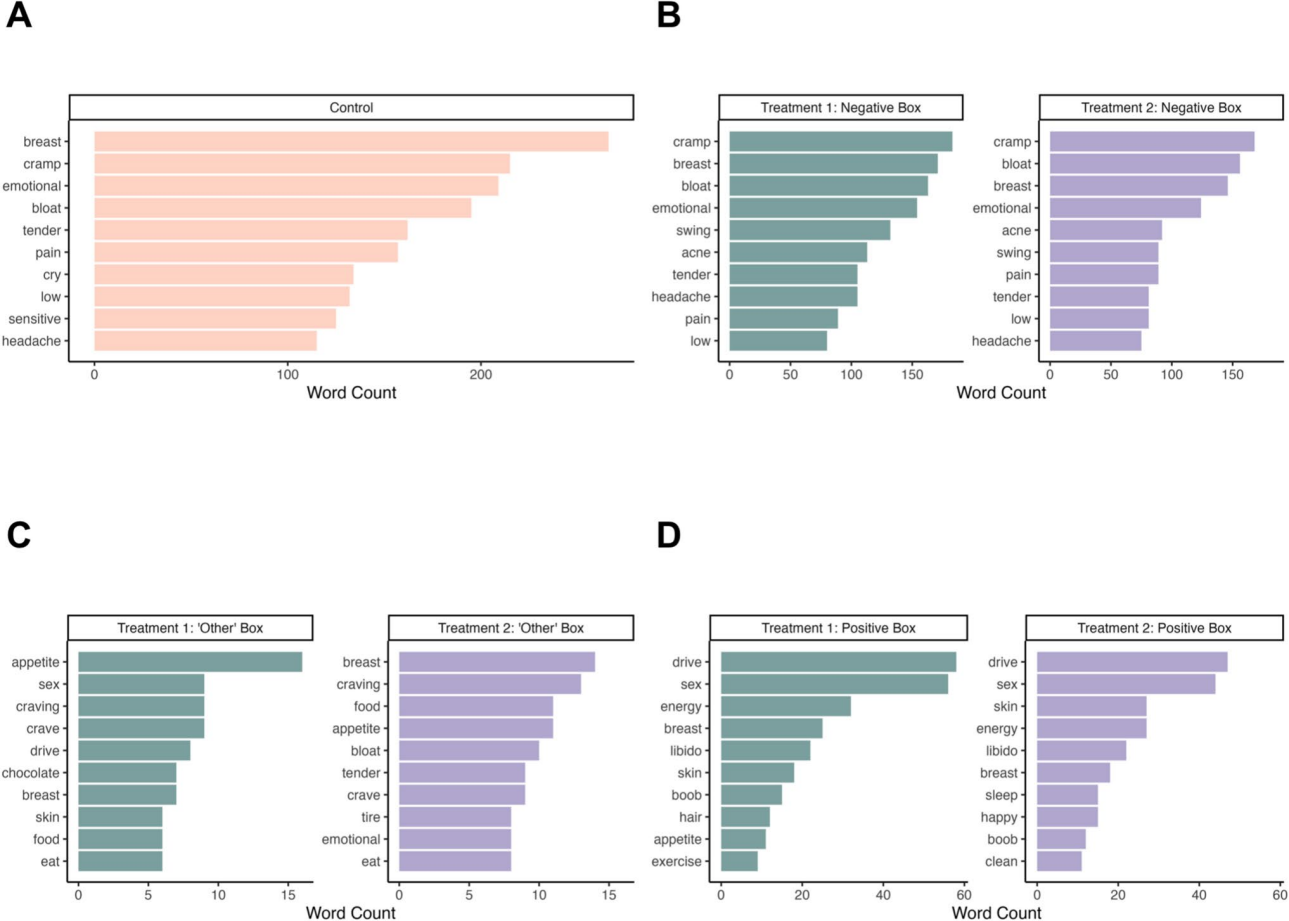
The ten most frequent words found in each condition. Control (A) contains mainly negative words at highest word counts. Treatment 1 and 2 top words in the negative (B) box are ‘cramp, breast, bloat’, while their top ‘other’ box content (C) focuses on food and cravings. Treatment 1 and 2 top positive (D) words are ‘sex’ and ‘drive’. Many words such as ‘breast’, ‘cramp’ and ‘bloat’ are found across all three conditions, while the order of these varies slightly between the conditions

The top ten most common words in the control condition were also the most common words in the negative boxes of treatment conditions, including tender, breast, cramp, emotional, bloat, pain and low. The word ‘cry’ was exclusively present among the top ten most frequent words in the control condition. Conversely, swing and acne were found only in the negative boxes of treatment conditions (Fig. 4). Breast was the only frequent word common to both the positive boxes in the treatment conditions and the control condition’.

##### Bigrams

**Fig. 5:**
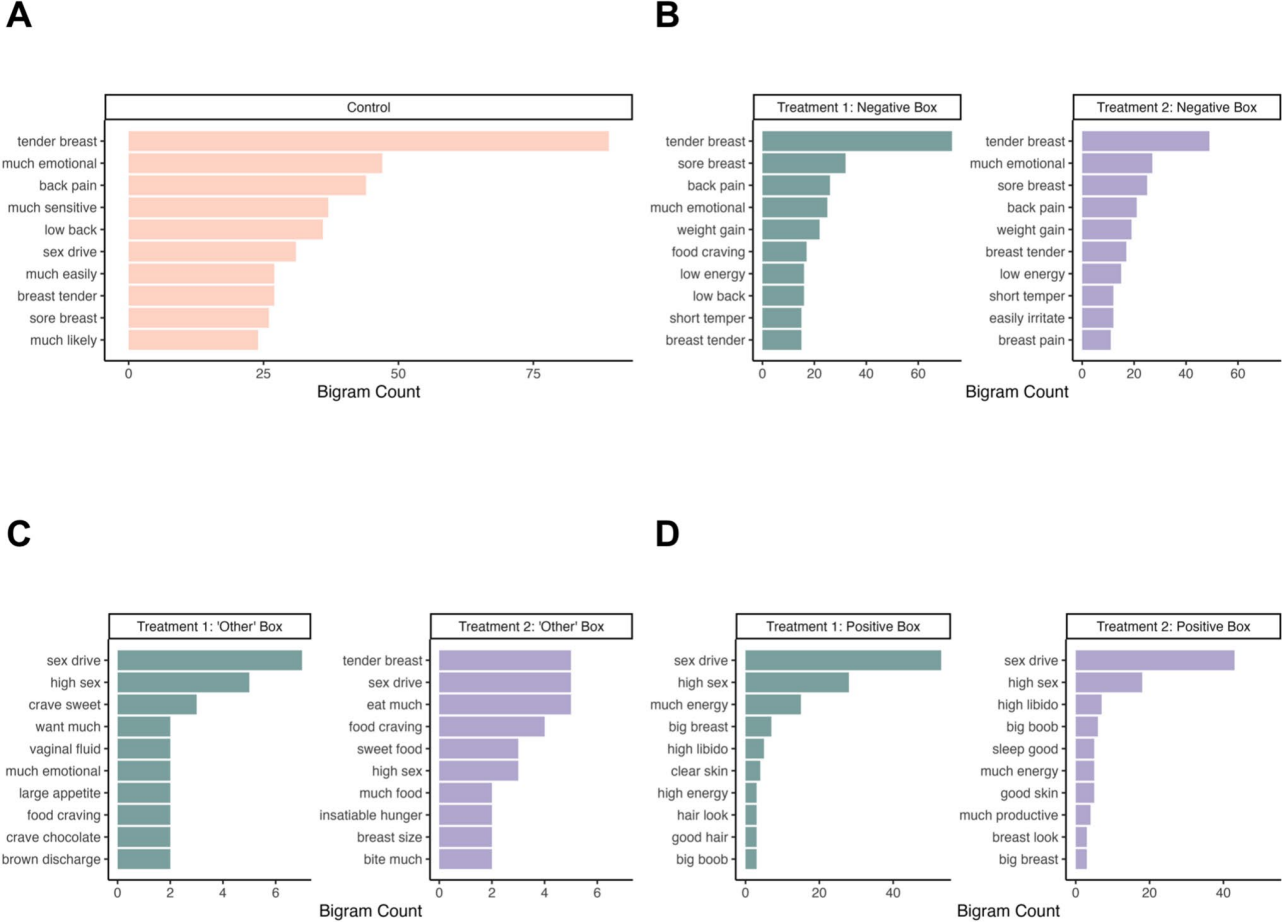
The ten most frequent bigrams found in each condition split by response box type. Control (A) contains mainly negative bigrams at highest word counts. Treatment 1 and 2 top bigrams in the negative (B) box are pain related, while their top ‘other’ box content (C) focuses on food, and cravings. Treatment 1 and 2 top positive (D) bigrams are focused on libido and energy. Some bigrams such as ‘tender breast’, ‘back pain’ and ‘sex drive are found across all three conditions, while the order of these varies slightly between the conditions

We found both similarities in bigrams (pairs of adjacent words) in the control condition and the negative boxes of the treatment conditions (e.g., ‘tender breast’, ‘back pain’, and ‘much emotional’ (Fig. 5)) as well as differences, for example the treatments had descriptions of increased energy and improved skin.

#### Most unique words across conditions

We observed that lexical density scores, calculated as the ratio of unique words to the total number of words within each condition, were highest in treatment conditions compared to the control condition (Fig. 3C). The diversity of words used was not influenced by the order of the emotional prompts.

#### Bias introduced by survey design

While the word count was higher in the control condition, the lexical density score were still higher in the treatment conditions despite expandable boxes being initially smaller. The control condition had a significantly higher word count per participant compared to the treatment conditions; no significant difference was found between the treatment conditions (Kruskal–Wallis ${\chi }^{2}$=361.57, df = 2, p < 0.001). There were no differences in the time taken to complete the questionnaire (Kruskal–Wallis ${\chi }^{2}$=5.94, df = 2, p < 0.001, Table 1).

## Discussion

We aimed to capture internalized views of the premenstrual experience (PME) by collecting online large open-ended text data in the absence and presence of emotional prompts. The findings show that reports of PME are somewhat malleable and hold implications for the measurement of the premenstrual experience.

### Premenstrual testimonies are negative overall

The premenstrual experience is predominantly characterized by negative accounts. These results align with prior research (Stubbs and Costos 2004; Ussher and Perz 2020). More than 70% of the scores across all three conditions were categorized as negative, and, strikingly, almost half of the responses from the treatment conditions exclusively filled the ‘negative’ box. We observed a consistent pattern across all three conditions; the most common words primarily revolved around themes of pain, emotions, and sensitivity, which may suggest the presence of biological factors contributing to negative premenstrual experiences. One possible explanation lies in the inflammatory response triggered by falling progesterone levels prior to menstruation (Bertone-Johnson et al. 2014; Alvergne and Högqvist Tabor 2018; Gold et al. 2016).

We observed differences in how negative responses were across conditions. In the absence of emotional prompts, the tendency to provide negative testimonies and the intensity of these negative testimonies were more pronounced. Positive responses were not more positive in the presence of emotional prompts; instead, negative responses were less negative. The lower negative emotional intensity seen in the treatment conditions may indicate that individuals opt for a neutral response to avoid the cognitive effort needed to provide a polarized response (Krosnick et al. 2001) with increasing options (Weijters et al. 2010).

### Positive symptoms are driven by energy and sex drive

The treatment conditions yielded a higher number of positive responses, which were mainly centered around testimonies concerning sex drive and libido. At the same time, the treatment conditions also highlighted energy and positive effects on hair, skin, and breasts. This aligns with research in the advertising industry, which has demonstrated that framing can influence responses and behavior (Chang 2008).

### Question phrasing influences writing style and text diversity

The word count was higher in the control condition, which may be due to the larger text box(Smith 1993; Christian and Dillman 2004; Smyth et al. 2009), but the treatment conditions showed a higher lexical diversity, i.e., a broader range of responses with fewer word repetitions. The control condition’s responses were more verbose, characterized by longer sentences with more descriptive words. The disparity in word density between conditions cannot be solely attributed to the number of response boxes since the control condition hadonly one text box, yet nearly double the word count compared to the treatment conditions. The responses in the treatment conditions were organized as lists of symptoms, which contributed to the differences in lexical density. The way responses were elicited and categorized influenced the participants’ writing style and the richness of their word choices.

Treatment conditions had a higher occurrence of neutral responses than the control condition, primarily stemming from statements such as “I don’t get PMS” or “none.” Unlike some previous studies (Romans et al. 2013; Kues et al. 2018), we did not find any significant impact of question wording order on the responses, possibly because participants in our study were able to view all the words within the same question, while other studies administered separate questionnaires for positive and negative surveys (Chrisler et al. 1994).

### Strengths

The inclusion of text boxes with explicit prompts for various experiences provided women with a platform to express their thoughts and concerns freely. Using computational methods also provided the opportunity to encompass a large volume of experiences, overcoming the limitations associated with conventional focus conditions and interviews.

## Limitations

First, we used an adapted dictionary lookup for text analysis because no annotated training data was available for female or reproductive health. Only a few studies have used deep learning to address issues of cross-domain sentiment classification (Moslmi et al. 2017) and creating a more advanced model was beyond the scope of this study. Note that sentiment analysis yielded reasonably accurate results on the responses, achieving over 75% accuracy for positive responses and over 85% accuracy for negative responses. Second, reports are based on retrospective recalls of premenstrual experiences, which can introduce bias. Although daily self-reports are recommended to reduce bias, the challenge of maintaining such diaries leads to dropout rates of up to 50%, leading to response bias (Sternfeld et al. 2002). Some research has shown no distinction in premenstrual ratings between retrospective and prospective collection methods (Steiner and Streiner 2005). Third, data on the duration of contraceptive use are not available, which could explain why contraceptive use is not associated with variation in emotional polarity. Finally*,* our approach does not account for cultural variation in the experience of PMS, nor does it consider group-specific differences in understanding menstrual-related terms. However, it provides an overview of the variation in premenstrual experiences and the impact of phrasing on their description. Future studies would benefit from investigating how sentiments related to reproductive health terms vary across cultural norms.

## Conclusion

By default, positive premenstrual experiences are not reported, unless specifically asked for, an effect we argue results from the influence of language and social constructs on how the premenstrual experience is described. Re-framing the discussion of the premenstrual phase as encompassing more than simply ‘negative symptoms’ could help de-construct the concept of PMS, and reduce the negative connotations that it invokes (Richardson 1995; Bancroft 1995).

## Data Availability

The data supporting the findings of this study are publicly available on the Open Science Framework (OSF) at [https://osf.io/mh2yx/?view_only=1df10f0c2fc94f44a59204ce74299946].
